# Postpartum Retroperitoneal Fibrosis With IVC Obstruction: A Case Report

**DOI:** 10.1155/crra/6285423

**Published:** 2026-07-01

**Authors:** Roy Clark Basiimwa, Samuel Bugeza, Rita Nassanga, Dell P. Dunn

**Affiliations:** ^1^ Department of Radiology, School of Medicine, Makerere University, Kampala, Uganda, mak.ac.ug; ^2^ Department of Radiology, University of Utah Hospital, Salt Lake City, Utah, USA; ^3^ Department of Radiology, Salt Lake City VA Medical Center, Salt Lake City, Utah, USA

**Keywords:** autoimmune disease, postpartum, retroperitoneal fibrosis

## Abstract

Retroperitoneal fibrosis (RPF) is a rare fibroinflammatory condition producing inflammatory masses in the retroperitoneum that can obstruct retroperitoneal structures, including vessels and ureters. RPF typically presents in middle‐aged men and may be associated with IgG‐4–related disease (IgG‐4 RD). This report describes a 34‐year‐old woman who presented 6 months postpartum with left leg swelling and lower abdominal pain. Contrast‐enhanced CT revealed a homogenous retroperitoneal soft‐tissue mass encasing the aorta and iliac arteries, with inferior vena cava (IVC) obstruction and collateral venous return through extensive pelvic varices. MRI findings included hypointensity on T2‐weighted imaging and restricted diffusion. Management with thrombolytics and steroids resulted in recanalization of the IVC, though persistent hydronephrosis required nephrostomy and further urological intervention. The presentation of RPF in a young woman is unusual, and we theorize that immune system reactivation following the relative immunosuppression during pregnancy may have precipitated the condition. The case illustrates one of several immunologic factors associated with RPF.

**Takeaway Lessons**

This case provides radiological insight into the diagnosis of retroperitoneal fibrosis (RPF), particularly in an atypical demographic, that is to say, postpartum women. Radiologists should note the utility of contrast‐enhanced CT for characterizing RPF, identifying characteristic features such as homogeneous retroperitoneal masses encasing the aorta and iliac vessels, medialization of the ureters, and assessing vascular complications, such as inferior vena cava (IVC) obstruction.

**Objectives**

• To demonstrate the multimodal radiological features of RPF in an atypical patient.
• To propose postpartum immune changes as a potential cause of RPF in an atypical patient.

## 1. Introduction

RPF is a rare fibroinflammatory condition defined by the excess fibrous tissue in the retroperitoneal space [1, 2]. This can cause encasement of abdominal vasculature, most commonly the abdominal aorta or common iliac arteries [2, 3]. RPF also leads to blockage of the ureters, resulting in hydronephrosis and eventually kidney failure [2]. The estimated prevalence is 1–1.4 per 100,000 individuals [3, 4], with a peak incidence between the ages of 40 and 60 and a 2:1 male predominance [4, 5]. RPF may be idiopathic, which is historically termed Ormond′s disease, or secondary to IgG4‐related disease (IgG4‐RD), medications, malignancy, or other inflammatory conditions [1, 3].

The diagnosis of RPF is made using laboratory tests and radiological studies [4]. Laboratory tests like erythrocyte sedimentation rate and C‐reactive protein levels are nonspecific [3, 4]. Contrast‐enhanced CT is the primary modality, typically showing a homogeneous retroperitoneal soft‐tissue mass encasing the aorta and iliac arteries [1, 3–5]. MRI demonstrates disease activity with diffusion‐weighted imaging (DWI) sequences used to differentiate between chronic RPF, active RPF, and malignant retroperitoneal lesions [3, 4, 6, 7]. A biopsy could be required to rule out malignancy in certain presentations or if the mass does not respond to treatment [1, 2].

The management of RPF depends on its severity and location [8, 9]. Initial treatment commonly involves anti‐inflammatory medications or corticosteroids [4, 8]. In cases where medication does not work or when there is severe urinary tract obstruction, interventional radiological or surgical treatment may be needed [8].

This report demonstrates an unusual case of RPF presenting in a 34‐year‐old postpartum woman with complete IVC obstruction, which is an atypical demographic and a rarely described vascular complication, and discusses the potential role of postpartum immune modulation in the development of fibroinflammatory disease.

## 2. Case Presentation

A 34‐year‐old woman with no significant prior medical history presented in July 2023, 6 months postpartum, with progressive swelling of the left leg and lower abdominal pain. The patient′s relevant laboratory investigations, including inflammatory markers and serum IgG4 levels, were performed at the time of presentation. However, these records are no longer available despite efforts to retrieve them. This represents a limitation of this case report as this would have confirmed or excluded IgG4‐RD.

Contrast‐enhanced abdominopelvic CT performed at presentation demonstrated a homogeneous, well‐defined enhancing retroperitoneal soft‐tissue mass. It extended from below the renal vessels to the aortic bifurcation (Figure 1). The mass encased the aorta and bilateral common iliac arteries without marked vascular displacement (Figure 1). Complete obstruction of the IVC was identified (Figure 2), with compensatory collateral venous return through extensive pelvic varices draining via the gonadal veins (Figures 3 and 4) into the left renal vein and the suprarenal IVC (Figure 5). Right ureteral obstruction with resultant hydronephrosis and hydroureter was also present (Figure 5). Notably, the pancreas and biliary tree appeared normal, and no renal deposits or discrete abdominal masses were identified, making IgG4‐related organ involvement and malignancy less likely. One week after the CT scan, a digital subtraction venogram was performed under fluoroscopy. This was to confirm complete IVC obstruction with collateral shunting through the left gonadal vein. These images were not available from the patient. Attempts at IVC recanalization at this time were unsuccessful.

**Figure 1:**
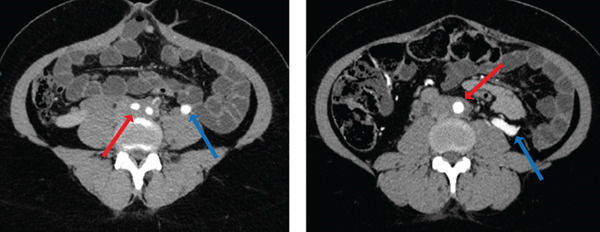
Axial contrast‐enhanced CT images in the arterial phase show a homogeneous retroperitoneal mass. This mass encases the aorta and common iliac arteries (red arrows). A large left gonadal vein is opacified (blue arrows).

**Figure 2:**
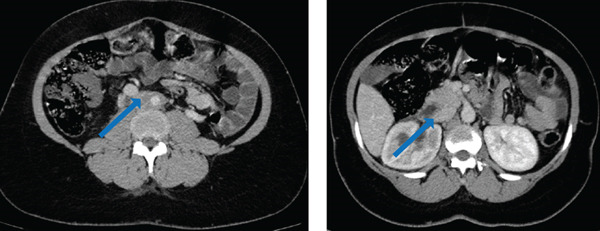
Axial contrast‐enhanced CT images in portal venous phase showing nonenhancement of the IVC below and enhancement above the level of the renal vessel (blue arrows).

**Figure 3:**
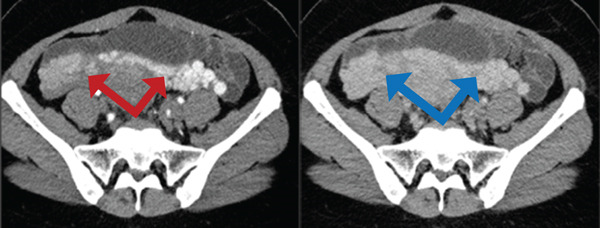
Axial contrast‐enhanced CT images in arterial (red arrows) and portal venous (blue arrows) phases show extensive opacified pelvic varices.

**Figure 4:**
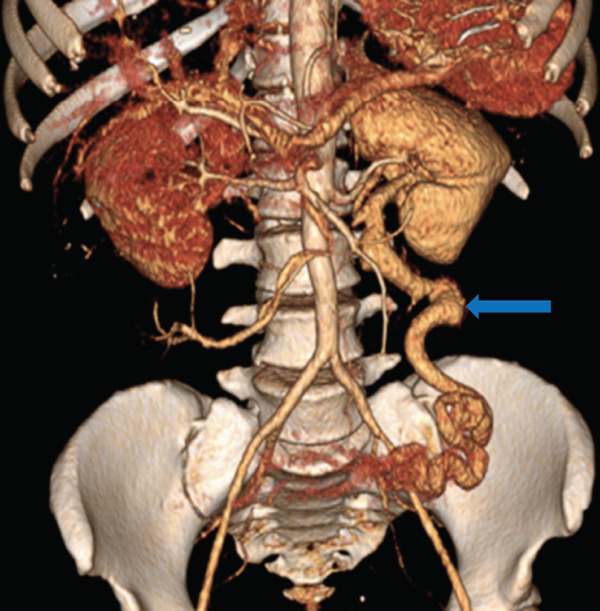
A 3D reconstructed CT image showing an enlarged left gonadal vein (blue arrow) draining into the retroaortic left renal vein and non‐opacification of the inferior vena cava.

**Figure 5:**
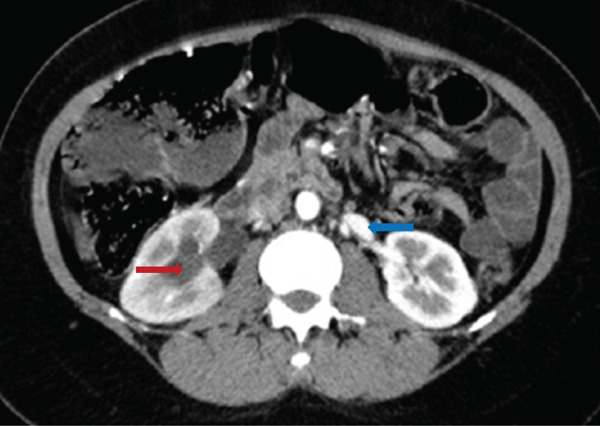
Axial econtrasted‐enhacned CT image in arterial phase showing the left gonadal vein (blue arrow) opacified in an arterial enhancement pattern showing collateral flow in the presence of IVC obstruction. There is hydronephrosis and hydroureter of the right kidney (red arrow).

With the diagnosis of RPF with IVC obstruction and right hydronephrosis, the patient was initiated on thrombolytics and corticosteroids. A percutaneous nephrostomy tube was inserted shortly thereafter to treat the right‐sided hydronephrosis. Despite 4 weeks of conservative management, the right ureteral obstruction persisted. A follow‐up antegrade pyelogram (Figure 6) through the nephrostomy tube shows medialization of the right ureter with mid‐ureteral obstruction, requiring further urological intervention.

**Figure 6:**
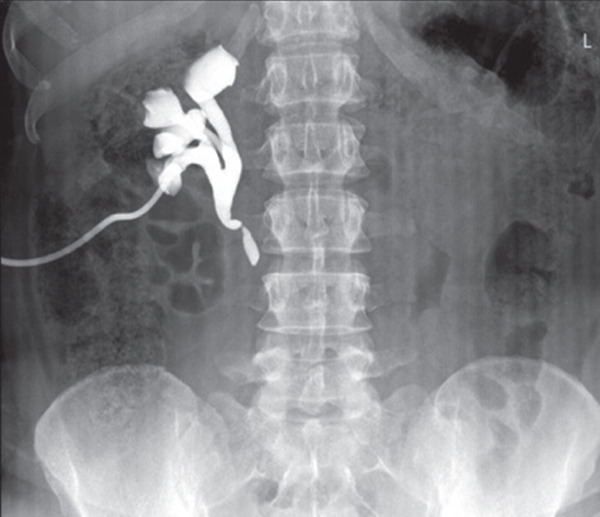
An antegrade pyelogram of the right kidney through the nephrostomy showing medialization of the right ureter with obstruction in the mid‐ureter.

An MRI was performed following 3 months of conservative therapy. T2‐weighted imaging demonstrated a hypointense fibrous retroperitoneal mass encasing the aorta (Figure 7). DWI with apparent diffusion coefficient (ADC) mapping showed restricted diffusion within the mass, indicating active disease (Figure 8).

**Figure 7:**
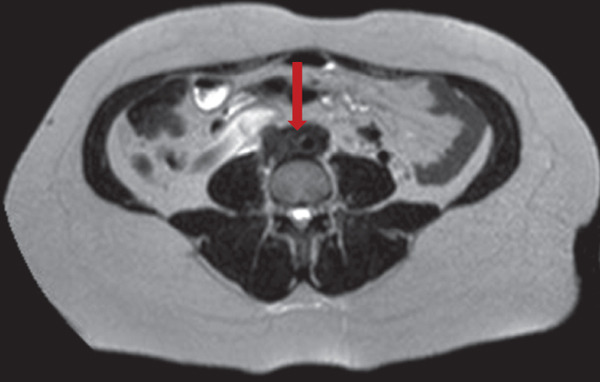
A T2‐weighted MR image showing retroperitoneal fibrosis as a hypointense fibrous mass (red arrow) surrounding the aorta.

**Figure 8:**
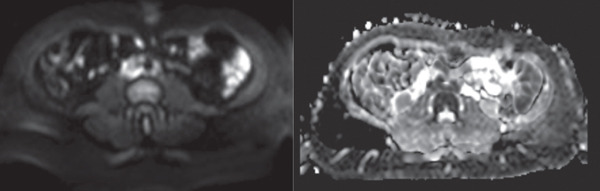
Mass demonstrates restricted diffusion with increased signal on high B‐value DWI and low signal on ADC map indicating active RPF.

## 3. Discussion

This case demonstrates the diagnostic problems of RPF in an unusual patient (a young postpartum woman). RPF is most commonly seen in middle‐aged to older males and often presents secondary to idiopathic processes, IgG4‐related disease, inciting medications, or malignancy. The patient′s age, sex, and postpartum status made this an uncommon presentation, and the additional finding of complete IVC obstruction with extensive collateral venous drainage further distinguished this case from typical RPF presentations.

The imaging findings in this case are characteristic of RPF, presenting as a fibrous, homogeneous mass encasing the aorta and iliac vessels without notable displacement, consistent with established imaging descriptions in the literature [8, 9]. Although vascular encasement is a well‐recognized feature of RPF, complete IVC obstruction with extensive pelvic collateralization is less commonly described, and this case demonstrates the importance of assessing venous structures in suspected RPF cases [7–9].

Several retroperitoneal conditions mimic RPF on imaging, and identifying them distinctly is important for management. Lymphoma is the main differential diagnosis to consider, as it also presents as a bulky retroperitoneal soft‐tissue mass. A useful imaging distinction is that lymphoma tends to push the aorta forward, whereas RPF does not. Lymphoma also rarely obstructs vessels or ureters, which is much more common in RPF [3, 9]. Malignancy‐associated retroperitoneal disease typically shows irregular margins, uneven enhancement, and invasion into surrounding tissue, unlike the smooth, uniform appearance of idiopathic RPF [1, 5]. Sarcomas appear as large, irregular masses with areas of necrosis or bleeding, whereas metastatic disease typically presents as multiple distinct lesions with a known primary cancer [1, 3]. In this case, the uniform enhancement pattern, absence of discrete masses, and normal appearance of the pancreas, biliary tree, and kidneys made malignancy unlikely. MRI further supported active RPF, with T2 hypointensity reflecting dense fibrous tissue and restricted diffusion on DWI signifying active disease [6, 7].

Using T2‐weighted imaging with DWI, tissue composition and disease activity of the differential diagnoses are radiologically differentiated. Hypointense T2 signal indicates fibrous tissue, whereas hyperintense tissue indicates active inflammation [6, 7]. DWI provides information on restricted diffusion; high B‐value with low ADC depicts active inflammation [6, 7]. In this case, T2 hypointensity with restricted diffusion on DWI matches with fibrous tissue with active inflammation. This is consistent with active RPF [6, 7]. Table 1 provides a comparative summary of the radiological findings of the differential diagnoses discussed above:

**Table 1:** Image‐based comparison of the differential diagnoses of idiopathic RPF.

Diagnosis	Radiological findings	Comparison to RPF
Lymphoma	Bulky, lobulated retroperitoneal soft tissue mass; heterogeneous enhancement and retroperitoneal lymphadenopathy; may encase vessels; hyperintense on DWI	Displaces aorta anteriorly; does not medialize the ureter; responds to chemotherapy rather than steroids; hyperintense on T2WI
Retroperitoneal sarcoma	Large heterogeneous mass with areas of necrosis; lobulated margins, calcifications; hyperintense on DWI	Heterogeneous rather than homogeneous in RPF; mass displaces rather than encase vessels; lobulated margins; hyperintense on T2WI
Metastatic disease	Nodal retroperitoneal masses usually with known primary; heterogeneous with central necorsis; hyperintense on DWI	Usually nodal masses rather than a confluent mass; no encasement of vessels; hyperintense on T2WI

The postpartum period is characterized by significant immune changes, hormonal shifts, and vascular remodeling that may contribute to the development of fibroinflammatory conditions [10]. In pregnancy, the immune system adjusts to promote fetal tolerance, creating an environment that suppresses inflammatory responses [10]. After childbirth, the immune system reverts to its pre‐pregnancy state, which may occasionally lead to immune dysregulation and may cause inflammatory or autoimmune conditions in individuals with a predisposition [10, 11]. The presence of fetal cells in maternal tissues has also been suggested as a possible immune trigger, potentially contributing to the chronic inflammation underlying RPF, though further studies are needed to confirm this link [10].

IgG4‐related disease is linked to a significant proportion of RPF cases and should be considered in all presentations, including unusual ones [8]. On imaging, IgG4‐related RPF appears identical to idiopathic RPF, presenting as a homogeneous enhancing mass encasing vascular structures. Other abdominal findings, such as autoimmune pancreatitis, bile duct thickening, and renal deposits, can suggest IgG4‐related etiology, none of which were present in this case [8]. Although serum IgG4 levels were unavailable for this patient, the autoimmune hypothesis is relevant given her postpartum context, in which immune dysregulation may have predisposed her to IgG4‐related or other autoimmune processes [8, 10].

Initial management of RPF commonly involves corticosteroids and anti‐inflammatory medications [4, 8]. When medications are ineffective, interventional treatment may be needed [8]. In this patient, thrombolytics and corticosteroids resulted in IVC recanalization, although persistent right ureteral obstruction required nephrostomy tube placement and further urological intervention.

## 4. Conclusion

This case shows an unusual presentation of RPF in a postpartum woman with complete blockage of the IVC. RPF commonly occurs in middle‐aged men, making this case rare and notable. The theory this case poses is that postpartum immune changes can contribute to the development of RPF in an atypical patient. More research is needed to link these changes to RPF.

## Author Contributions

All authors participated in writing/editing, figure collection, and reviewing the case report.

## Funding

No funding was received for this manuscript.

## Consent

Ethics approval is not required for case reports at our local hospital Institutional Review Board. Written informed consent was obtained from the patient.

## Conflicts of Interest

The authors declare no conflicts of interest.

## Data Availability

The data that support the findings of this study are available from the corresponding author upon reasonable request.
